# Considerations About the Antimicrobial Activity of Oxidized Cellulose and Oxidized Regenerated Cellulose and Their Potential Application in Veterinary Surgery

**DOI:** 10.3390/vetsci13040349

**Published:** 2026-04-03

**Authors:** Riccardo Rinnovati, Paola D’Angelo, Angelo Peli, Maria Virginia Ralletti, Federica Meistro

**Affiliations:** 1 Department of Veterinary Medical Science, University of Bologna, Ozzano dell’Emilia, 40064 Bologna, Italy; riccardo.rinnovati2@unibo.it (R.R.); virginia.ralletti@unibo.it (M.V.R.); federica.meistro@unibo.it (F.M.); 2 Department for Life Quality Studies, University of Bologna, 47291 Rimini, Italy; angelo.peli@unibo.it

**Keywords:** antimicrobial activity, bioabsorbable hemostatic agents, infection control, oxidized cellulose, oxidized regenerated cellulose, surgical site infection, veterinary surgery

## Abstract

Oxidized cellulose (OC) and oxidized regenerated cellulose (ORC) are bioabsorbable polysaccharide-based materials widely used in surgery for topical hemostasis. Beyond their established hemostatic role, increasing attention has been directed toward their potential antimicrobial activity, primarily attributed to local acidification following carboxyl group dissociation. Discussing the possible implications of the antibacterial properties of OC/ORC in veterinary surgical practice, this review critically examines the existing evidence. In vitro studies show that viable bacterial counts are significantly reduced in both Gram-positive and Gram-negative pathogens, including antibiotic-resistant strains. Historical in vivo animal models further support a reduction in bacterial recovery in contaminated tissues treated with OC. However, contemporary veterinary clinical trials specifically evaluating surgical site infection (SSI) outcomes remain limited. Documented limitations include variability in formulation, quantity-dependent degradation kinetics, and the potential for foreign body reactions when excessive material is retained. Current evidence suggests that OC may provide adjunctive antimicrobial effects under controlled experimental conditions, primarily in vitro and in standardized animal models, but these properties should be interpreted with caution, and its role should be integrated within comprehensive infection prevention strategies rather than considered a substitute for established perioperative protocols.

## Simple Summary

Oxidized cellulose (OC) and oxidized regenerated cellulose (ORC) are used in veterinary surgery as topical hemostatic agents. In addition to their role in controlling bleeding, these materials may also exert antimicrobial effects through local acidification. This potential dual action is of particular interest in veterinary surgical settings, where the risk of contamination and surgical site infection remains a clinical concern. This review critically examines the available evidence on the antimicrobial activity of OC/ORC and discusses their potential clinical relevance, limitations, and implications for veterinary surgical practice.

## 1. Introduction

Cellulose is a natural linear polysaccharide composed of β-(1→4)-linked anhydroglucose units [1] and represents the most abundant natural polymer on Earth [2]. Its chemical structure includes reactive hydroxyl groups that can undergo oxidation, leading to the formation of oxidized cellulose (also referred to as oxycellulose) [1,3,4]. This chemical modification significantly alters the biological behavior of the material [3].

Several oxidation pathways of cellulose have been described, depending on the oxidizing system and reaction conditions, leading to different chemical structures and material properties [3,4,5]. However, in clinical applications, oxidized cellulose is typically produced through selective oxidation of primary hydroxyl groups at the C6 position, most commonly using nitrogen oxides, resulting in carboxyl-rich materials such as oxidized regenerated cellulose. At the molecular level, oxidation predominantly targets the primary hydroxyl groups at the C6 position of the anhydroglucose units, although secondary hydroxyl groups at C2 and C3 may also be involved depending on the oxidizing system employed [6]. Consequently, a heterogeneous distribution of functional groups can be generated along the polymer chain, primarily consisting of carboxyl groups, although aldehyde and ketone moieties may also be present depending on the oxidation conditions [3,4,5].

Unlike native cellulose, oxidized cellulose is biodegradable and resorbable in vivo, properties that have supported its clinical use as a topical hemostatic agent since the mid-20th century [1]. The introduction of carboxyl groups into the cellulose structure not only enhances its hemostatic capacity but also confers antimicrobial activity, mainly through the rapid local acidification of the surrounding environment [5]. This dual action, mechanical hemostasis, combined with a localized reduction in bacterial growth, makes oxidized cellulose particularly attractive in surgical settings where bleeding control and contamination risk coexist [1,7].

### 1.1. Differences Between OC and ORC

A technical distinction should be made between oxidized cellulose (OC) and oxidized regenerated cellulose (ORC), as they differ in production process and structural organization. Native oxidized cellulose is obtained through direct oxidation of natural cellulose fibers, whereas ORC is produced via a preliminary dissolution and regeneration step, resulting in a more uniform structure and more controlled physicochemical properties [5,8]. This leads to a more organized matrix with more predictable degradation behavior. Additionally, oxidation of regenerated cellulose, typically performed using nitrogen oxides, promotes a more homogeneous distribution of functional groups and reduces variability in material performance [9]. Comparative investigations have shown that non-regenerated oxidized cellulose is characterized by a more irregular and frayed fiber structure, providing greater surface area, whereas ORC displays a smoother and more organized architecture. These structural differences may influence several functional properties, including local pH behavior; ORC has been reported to generate a more pronounced initial acidification under such conditions, particularly in low-buffered environments such as saline or phosphate-buffered solutions, where there is limited buffering capacity, which allows a more pronounced local acidification. In contrast, these differences tend to diminish in protein-rich and physiologically buffered fluids such as plasma. Hemostatic efficiency has been reported to be superior in non-regenerated forms. In contrast, with respect to the focus of this review, bactericidal activity appears largely comparable between the two materials, suggesting that antimicrobial effects are primarily driven by local acidity rather than fiber organization alone [7].

### 1.2. Rationale in Veterinary Surgery

The occurrence of surgical site infections (SSIs) in veterinary medicine remains a significant concern, and OC could play a role in addressing this issue. SSIs affect both small animal and large patients, with reported incidences ranging from less than 1% to over 20% depending on the type of procedure and perioperative management, and are associated with prolonged hospitalization, increased treatment costs, delayed wound healing, and, in severe cases, life-threatening complications [10]. In this context, biomaterials that combine hemostatic and antimicrobial properties may represent a valuable tool in veterinary surgical practice [11]. While oxidized cellulose has been widely adopted in human surgery [7,12], its potential antimicrobial role in veterinary medicine has received comparatively limited attention, where its application remains largely confined to bleeding control. Although several studies have investigated the physicochemical properties and antimicrobial activity of oxidized cellulose, most of the available evidence originates from in vitro experiments or human medical research, and only a limited number of studies have addressed its role in veterinary surgical practice. Consequently, available evidence of its antimicrobial properties remains fragmented, highlighting the need for a critical, veterinary-focused evaluation of its antimicrobial relevance.

In light of this, the aim of the present review is to critically assess the current evidence on the antimicrobial properties of oxidized cellulose and to examine its potential clinical relevance, advantages, and limitations within veterinary surgical practice.

## 2. Methods

This study was conducted as a narrative literature review. A structured search of PubMed, Scopus, and Google Scholar databases was performed to identify publications addressing oxidized cellulose (OC) and oxidized regenerated cellulose (ORC) in relation to antimicrobial activity, biomaterial behavior, and surgical applications. Articles published between 1970 and 2025 were considered, including experimental studies, clinical reports, and relevant reviews. Given the limited veterinary-specific evidence, human medical literature was included to provide a mechanistic and translational context. Studies were qualitatively analyzed based on relevance and scientific consistency.

## 3. Mechanism of Antimicrobial Activity

**Figure 1:**
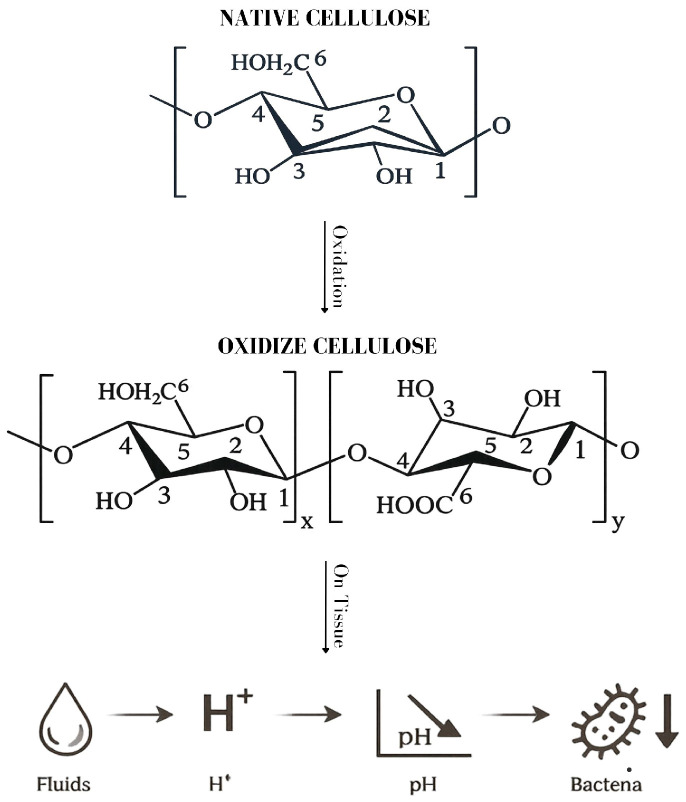
Overview of the chemical modification of native cellulose to OC and the proposed mechanism underlying their antimicrobial activity.

The antimicrobial properties of oxidized cellulose (OC) are not intrinsic to native cellulose but arise from its chemical modification during the oxidation process (Figure 1). The structural modification profoundly alters the physicochemical behavior of the material [1,3,4].

When oxidized cellulose comes into contact with blood, tissue fluids, or aqueous media, the introduced carboxyl groups dissociate, releasing hydrogen ions into the surrounding environment. As a result, a rapid and localized decrease in pH occurs at the material–tissue interface [7,13]. Aqueous suspensions of oxidized cellulose can reach pH values of approximately 2 to 4, and this acidic environment may persist for several hours, even in the presence of biological fluids [11,14]. This localized acidification is considered the primary mechanism underlying the antimicrobial activity of oxidized cellulose [7,13,14].

Most clinically relevant bacterial pathogens are considered neutralophilic organisms, meaning they grow best in near-neutral external pH conditions and maintain their cytoplasmic pH within a narrow neutral range [15]. In small animal surgery, organisms such as *Staphylococcus pseudintermedius*, *Staphylococcus aureus*, *Staphylococcus epidermidis* and *Enterococcus* spp. are frequently reported [16,17], while in equine surgery *Staphylococcus* spp., *Streptococcus* spp., *Escherichia coli*, *Actinobacillus* spp., and occasionally *Pseudomonas aeruginosa* are commonly involved [18,19]. Exposure to markedly acidic conditions interferes with essential bacterial processes, including membrane integrity, enzyme activity, nutrient transport, and DNA replication. Sustained exposure to low pH may result in growth inhibition or cell death, depending on the organism and environmental conditions [15,20,21]. In addition to its antimicrobial action, oxidized cellulose has been reported to reduce local inflammation and oxygen free radical activity, as well as to decrease edema and support wound healing processes [11].

Experimental in vitro studies provide quantitative support for the antimicrobial activity of oxidized cellulose and its regenerated derivatives, confirming that the acidification described above translates into measurable reductions in bacterial viability (Table 1).

One of the most frequently cited investigations is that of Spangler et al. [13], who evaluated the antimicrobial activity of oxidized regenerated cellulose in three different formulations: SURGICEL absorbable hemostat (ORC-R), SURGICEL Fibrillar absorbable hemostat (ORC-F), and SURGICEL NU-KNIT absorbable hemostat (ORC-N), using a standardized broth suspension model with serial quantitative cultures against clinically relevant pathogens, including methicillin-resistant *Staphylococcus aureus* (MRSA), methicillin-resistant *Staphylococcus epidermidis*, vancomycin-resistant *Enterococcus* spp., and other antibiotic-resistant isolates. The authors reported significant reductions in viable bacterial counts, often reaching or exceeding 3 log_10_ reductions within 24 h of exposure. These effects were accompanied by a marked decrease in broth pH, supporting the hypothesis that environmental acidification plays a central role in the observed antimicrobial activity [13].

Further in vitro evaluation was provided by Alhetheel et al. [24], who investigated a commercially available oxidized regenerated cellulose product (Regecel) against a panel of bacterial and fungal strains. The material demonstrated 100% growth inhibition against methicillin-resistant *Staphylococcus aureus* (MRSA), vancomycin-resistant *Enterococcus* (VRE), and penicillin-resistant *Streptococcus pneumoniae*, while also exhibiting complete inhibition of *Candida albicans* under the tested conditions. Additional Gram-negative organisms, including *Escherichia coli* and *Pseudomonas aeruginosa*, showed similarly high inhibition rates, indicating broad-spectrum antimicrobial activity [24].

Additional experimental evidence has been provided by studies evaluating oxidized cellulose materials with different chemical compositions and physical structures. Vytrasova et al. demonstrated that oxidized cellulose salts, including formulations incorporating silver and zinc ions, exhibited inhibitory activity against a wide spectrum of Gram-positive and Gram-negative bacteria, yeasts, and filamentous fungi using dilution and diffusion assays, suggesting that antimicrobial performance may also be modulated by ionic composition and material configuration [22].

Similarly, Moťková et al. reported rapid reductions in bacterial cell density following exposure to an oxidized cellulose hemostatic textile (OKCEL^®^ H-D), with several tested microorganisms showing decreases in multiple logarithmic orders within hours of contact [23].

In vitro evidence confirms broad-spectrum activity influenced by both oxidation degree and structural configuration. Further studies are needed to clarify its clinical relevance in vivo.

## 4. Evidence of Antimicrobial Property in Clinical Use

### 4.1. Human Medicine

In human medicine, oxidized regenerated cellulose (ORC), alone or combined with collagen and silver, has been extensively investigated in chronic and high-risk wound management. Beyond its hemostatic role, clinical observations suggest a potential contribution to infection control. Chronic wounds are characterized by persistent inflammation and elevated protease activity, which impair healing and favor microbial persistence. Collagen/ORC matrices can bind and inactivate matrix metalloproteinases and elastase while preserving growth factors, thereby promoting a more favorable wound environment and supporting tissue repair [25,26,27].

Clinical studies report improved wound progression and reduced signs of critical colonization when these materials are incorporated into standard care protocols [28]. In pressure ulcers and venous leg ulcers, reductions in protease activity (including elastase, plasmin, and gelatinase) have been associated with improved healing trajectories [29,30]. Randomized controlled trials in chronic wounds, including diabetic foot ulcers and venous leg ulcers, have shown improved healing outcomes or wound area reduction with collagen/ORC-based dressings, although results are sometimes not statistically significant or comparable in long-standing wounds [25,31]. However, the authors emphasized that high-quality randomized trials specifically designed to measure antimicrobial endpoints remain limited, and that the antimicrobial contribution of ORC is often inferred from clinical outcomes rather than directly quantified through microbiological analysis [28].

### 4.2. Veterinary Medicine

Despite the limited availability and the historical nature of veterinary experimental studies specifically evaluating oxidized cellulose (OC) as an antimicrobial agent [13,22], experimental animal studies provide relevant in vivo evidence supporting its infection-modulating capacity. In a canine splenotomy model challenged with intravenous *Staphylococcus aureus*, splenic sites treated with oxidized regenerated cellulose (ORC) demonstrated significantly lower bacterial recovery compared with absorbable gelatin sponge controls during the early postoperative period, and ORC-treated sites maintained persistently low bacterial counts over a 20-day observation period. The author concluded that ORC effectively reduced the bacterial population following experimental challenge, particularly during the initial phase of infection [32].

Comparative experimental studies have also evaluated the behavior of oxidized cellulose in contaminated wound models. In a subcutaneous animal model with standardized bacterial inoculation, oxidized cellulose was compared with microfibrillar collagen and demonstrated a lower incidence of infection, indicating a more favorable profile under contaminated conditions. Notably, the observed effects appeared to be influenced by the amount of material applied, with higher quantities associated with less optimal outcomes, highlighting the importance of appropriate intraoperative use [33].

Despite their historical nature, these experimental studies, often based on standardized bacterial challenge and quantitative microbial assessment, provide only limited and context-dependent indications of a potential antimicrobial effect under biologically relevant conditions. The lack of contemporary veterinary infection models specifically evaluating oxidized cellulose further underscores the need for renewed investigation within modern antimicrobial stewardship frameworks.

## 5. Additional Applications in Veterinary Surgery

In veterinary surgery, ORC operates through a multifaceted mechanism involving both physical and chemical processes. These include surface-mediated clot formation, swelling, and a gel-like transformation upon contact with blood, all of which contribute to hemostatic stability [34]. Experimental implantation studies demonstrated that oxidized regenerated cellulose functions as a temporary bioabsorbable tissue matrix. Following surgical placement in soft tissues of experimental animal models, the material undergoes progressive structural degradation accompanied by cellular infiltration and fibrovascular integration, ultimately leading to gradual resorption without the need for removal. These findings support the clinical practice of leaving ORC in situ after surgical application when removal could compromise local hemostasis [35]. In a controlled porcine model of splenic and hepatic resection, oxidized regenerated cellulose was applied to parenchymal bleeding surfaces and evaluated over a six-week follow-up period. The study reported effective hemostasis comparable to alternative cellulose-based agents, with no evidence of abscess formation, secondary infection, or clinically significant inflammatory reaction at necropsy. Histological examination demonstrated progressive material degradation associated with limited macrophage infiltration and absence of severe foreign-body granulomatous response, and complete resorption was observed within the observation period [36].

Oxidized regenerated cellulose (ORC) is primarily employed as a topical absorbable hemostatic agent for the control of capillary, venous, and parenchymal bleeding. In small animal surgery, its use has been documented during partial splenectomy procedures, where Surgicel^®^ was applied directly to the splenic parenchyma to achieve rapid hemostasis without the need for extensive suturing or cauterization. The material is placed over the bleeding surface, where it conforms to tissue contours and promotes clot formation, after which it may be left in situ due to its bioabsorbable properties [37].

In equine surgery, ORC is described as an adjunctive topical hemostatic option in procedures associated with significant hemorrhagic risk, including sinonasal and paranasal sinus interventions [38]. Its application in these contexts is intended to assist with intraoperative bleeding control in highly vascular regions where visualization and surgical precision may otherwise be compromised. Additionally, in equine cervical and soft tissue surgery, topical hemostatic materials such as oxidized cellulose are used to support local bleeding control in anatomically complex or infected areas [39].

Beyond its traditional use in fabric form, oxidized regenerated cellulose has been developed into alternative formulations such as absorbable hemostatic powders, expanding its applicability in minimally invasive and difficult-to-access surgical sites. In vivo experimental models, particularly in porcine liver bleeding models, have demonstrated that ORC-based powders can achieve rapid and effective hemostasis, particularly in cases of diffuse capillary or mild venous bleeding, outperforming other commercially available hemostatic agents in terms of time-to-hemostasis. The mechanism of action involves not only physicochemical interactions but also a mechanical effect, as the material absorbs blood, swells, and provides a scaffold for clot formation by trapping platelets and plasma proteins [40].

Overall, within veterinary surgical practice, the documented applications of ORC center on intraoperative bleeding control in both small animal and equine procedures, particularly in parenchymal organs and highly vascular soft tissues, while formal investigations of additional biological effects remain comparatively limited [37,38].

## 6. Limitations, Risks, and Safety Considerations

Although oxidized regenerated cellulose (ORC) demonstrates consistent antimicrobial activity in vitro and in experimental animal models [13,24,32], several important limitations must be acknowledged when considering its role in veterinary infection control.

First, most antimicrobial data derive from controlled laboratory conditions or historical experimental models rather than contemporary veterinary clinical trials. While in vitro studies report significant reductions in viable bacterial counts and experimental models demonstrate decreased bacterial recovery in contaminated tissues [32,33], robust prospective clinical studies evaluating surgical site infection (SSI) rates in veterinary patients remain lacking [28]. Therefore, the translation of laboratory findings into predictable clinical infection prevention cannot be assumed.

Second, antimicrobial efficacy appears to be strongly dependent on direct contact between the material and microorganisms. In clinical settings characterized by exudate, tissue debris, necrotic material, or biofilm formation, the antimicrobial performance of ORC may be reduced if intimate contact is not achieved. None of the available veterinary studies specifically evaluate the efficacy of ORC against established biofilms in vivo, which represent a major challenge in both small animal and equine surgery.

Third, while localized antimicrobial activity may contribute to early bacterial load reduction, ORC does not possess targeted bactericidal mechanisms comparable to systemic antibiotics. Its antimicrobial effect is physicochemical rather than receptor-mediated, and therefore may vary depending on bacterial species, inoculum size, local buffering capacity, and tissue perfusion [1,13]. This variability limits the predictability of its performance in heterogeneous surgical environments.

From an antimicrobial stewardship perspective, it is essential to emphasize that ORC should not be interpreted as an alternative to appropriate perioperative antibiotic protocols. The antimicrobial effect described in experimental models represents an adjunctive local phenomenon rather than a substitute for systemic therapy [28]. Overestimation of its infection-preventive capacity could lead to inappropriate reduction in established prophylactic measures.

The quantity of oxidized regenerated cellulose applied represents a clinically relevant variable. Some authors explicitly recommend using the minimal effective amount necessary to achieve hemostasis and caution against excessive packing of the material. The degree of material retention and resorption time have been reported to depend on the amount implanted, with larger quantities potentially persisting for extended periods before complete degradation [7,12].

Although oxidized regenerated cellulose is biodegradable and generally considered biocompatible, it can act as a foreign body and elicit granulomatous inflammatory reactions when retained in situ. Case reports have documented ORC-induced granulomas that mimic recurrent tumors or abscesses on imaging, resulting in diagnostic uncertainty and potentially unnecessary interventions [41,42]. In experimental animal models, ORC residues have persisted for extended periods and provoked histologically evident foreign body reactions, characterized by multinucleated giant cells and macrophage infiltration [43]. Even though these events are uncommon, they underscore the importance of using the minimal effective amount of ORC and of carefully documenting its intraoperative placement. In addition to granulomatous responses, a range of postoperative complications has been reported in clinical settings, including seroma formation, allergic reactions (such as dermatitis and localized hypersensitivity), and, more rarely, compressive phenomena or infection-related complications associated with incomplete bioabsorption. Notably, ORC can persist as a gelatinous mass after contact with blood and tissue fluids, and its resorption kinetics may vary depending on the amount applied and local tissue conditions, potentially prolonging its presence in vivo and increasing the likelihood of adverse tissue responses [44].

Finally, formulation variability may influence antimicrobial performance. Differences in fiber architecture, oxidation degree, and product configuration (e.g., gauze, fibrillar, knit forms) affect degradation kinetics and local environmental modification, potentially leading to inconsistent antibacterial outcomes across products [1,13].

Evidence from human medical literature has also addressed strategies to reduce complications associated with the use of oxidized regenerated cellulose (ORC). A clinical review highlighted that these adverse outcomes, including foreign body reactions and granuloma formation, are frequently related to suboptimal surgical use rather than intrinsic material toxicity. In particular, excessive application, retention of large amounts of material, and use in confined anatomical spaces were identified as key contributing factors. To mitigate these risks, the authors emphasize the importance of applying the minimal effective quantity required for hemostasis, avoiding unnecessary packing, and considering the removal of excess material once bleeding is controlled. Furthermore, accurate intraoperative documentation of ORC placement is recommended to prevent postoperative diagnostic uncertainty [45].

In summary, while oxidized regenerated cellulose exhibits demonstrable antimicrobial properties under experimental conditions, current veterinary evidence does not yet support its use as a standalone infection control strategy. Its role should be considered adjunctive, context-dependent, and should be integrated within comprehensive surgical infection prevention protocols.

## 7. Future Perspectives

Future investigations should move beyond experimental antimicrobial demonstrations and establish clinically meaningful endpoints in veterinary populations. Prospective, adequately powered trials assessing surgical site infection (SSI) incidence, bacterial burden, and wound healing outcomes are necessary to determine whether the physicochemical antimicrobial properties of oxidized regenerated cellulose (ORC) translate into measurable clinical benefit. Given the complexity of contaminated and high-motion surgical environments in both small animal and equine practice, species-specific studies are particularly warranted. Standardized protocols evaluating dosage, formulation, and placement techniques would improve comparability across studies and clarify formulation-dependent variability.

## 8. Conclusions

Current evidence suggests that oxidized cellulose and oxidized regenerated cellulose exhibit antimicrobial activity under experimental conditions, with consistent findings across multiple in vitro studies demonstrating a reduction in bacterial viability, including clinically relevant and resistant strains. These results provide a strong biological rationale for a potential role in modulating the local microbial environment in surgical settings.

However, most of the available data derive from controlled experimental conditions, and veterinary in vivo studies specifically assessing surgical site infection outcomes remain limited. Consequently, although the antimicrobial effect is well supported at a mechanistic and experimental level, its translation into consistent and predictable clinical benefit cannot yet be definitively confirmed.

At present, this antimicrobial property should be considered a potential adjunctive advantage, complementing the well-established hemostatic role of these materials, rather than replacing standard infection prevention strategies. In clinical practice, their use should remain guided by surgical indication, with careful and conservative application, including the use of the minimum effective quantity and attention to anatomical context.

Overall, oxidized cellulose represents a promising material with both hemostatic and potential infection-modulating properties. Future veterinary studies, particularly well-designed in vivo and clinical investigations, are warranted to clarify the extent to which these experimental findings translate into a measurable clinical benefit and to better define their role within surgical infection control protocols.

## Figures and Tables

**Table 1:** Experimental studies evaluating the antimicrobial activity of oxidized cellulose (OC) and oxidized regenerated cellulose (ORC). Summary of in vitro investigations reporting quantitative microbial reduction following direct exposure to oxidized cellulose-based materials, including different formulations and tested microorganisms.

Study	Material tested	Cellulose type/formulation	Experimental model	Microorganisms tested	Antimicrobial outcome
Spangler et al., 2003 [13]	SURGICEL^®^ absorbable hemostat (ORC-R); SURGICEL^®^ Fibrillar (ORC-F); SURGICEL^®^ NU-KNIT (ORC-N)	Oxidized regenerated cellulose (ORC) hemostats	Broth suspension challenge with quantitative culture (CFU counts at 0, 1, 6, 24 h)	Methicillin-resistant *Staphylococcus aureus* (MRSA); Methicillin-resistant *Staphylococcus epidermidis* (MRSE); Vancomycin-resistant *Enterococcus faecalis (VRE)*; Vancomycin-resistant *Enterococcus faecium (VRE)*; Penicillin-resistant *Streptococcus pneumoniae*; *Staphylococcus aureus*; *Pseudomonas aeruginosa*	≥3 log_10_ reduction for most tested strains within 24 h; antimicrobial activity strongly associated with acidic pH and reduced when neutralized; variable response observed in some VRE isolates.
Vytrasova et al., 2008 [22]	OKCEL^®^ H-L; OKCEL^®^ Zn-M; OKCEL^®^ ZnNa-L; OKCEL^®^ ZnNa-M; OKCEL^®^ Ag-L; textile forms (OKCEL^®^ Ag-T, Zn-T, H-T)	Oxidized cellulose salts (H+, Zn2+, Ag+, mixed salts) in linter, microsphere and textile structures	Dilution assay and diffusion method	*Escherichia coli*; *Pseudomonas aeruginosa*; *Staphylococcus epidermidis*; *Bacillus licheniformis*; *Clostridium perfringens*; *Candida albicans*; *Candida tropicalis*; *Aspergillus niger*; *Penicillium chrysogenum*; *Rhizopus oryzae*; *Scopulariopsis brevicaulis*	Strong inhibition of Gram+ and Gram− bacteria and fungi; Silver-containing formulations showed among the highest antimicrobial activity; Zn-modified materials inhibited multiple bacterial and fungal species
Moťková et al., 2017 [23]	OKCEL^®^ H-D absorbable hemostatic textile	Oxidized cellulose textile hemostat	Dilution suspension test and diffusion assay	*Arcanobacterium haemolyticum*; *Bacillus subtilis*; *Bacteroides fragilis*; *Moraxella catarrhalis*; *Clostridium perfringens*; *Corynebacterium xerosis*; *Enterobacter cloacae*; *Enterococcus faecalis*; *Escherichia coli*; *Klebsiella pneumoniae*; *Listeria monocytogenes*; MRSA; MRSE; *Mycobacterium smegmatis*; *Proteus mirabilis*; *Proteus* spp.; *Pseudomonas aeruginosa*; *Pseudomonas stutzeri*; *Salmonella Enteritidis*; *Serratia marcescens*; *Staphylococcus aureus*; *Staphylococcus epidermidis*; *Staphylococcus saprophyticus*; *Streptococcus agalactiae*; *Streptococcus pyogenes*; *Streptococcus salivarius*; Vancomycin-resistant *Enterococcus* (VRE)	Reduction in bacterial density up to 7–8 log_10_ after 6 h exposure for most organisms; inhibition zones observed for nearly all tested strains; weaker effect on spore-forming *Bacillus subtilis* and *Mycobacterium smegmatis*
Alhetheel et al., 2024 [24]	REGECEL^®^ (standard, knit, fibrillar, non-woven variants)	Commercial oxidized regenerated cellulose (ORC) wound dressing	Microbial challenge plate assay	*Pseudomonas aeruginosa*; *Enterobacter cloacae*; *Enterococcus faecalis*; *Klebsiella pneumoniae*; *Staphylococcus saprophyticus*; *Micrococcus luteus*; *Corynebacterium striatum*; *Escherichia coli*; *Salmonella enteritidis*; MRSA; *Streptococcus pneumoniae*; *Haemophilus influenzae*; *Streptococcus agalactiae*; *Candida albicans*; *Moraxella catarrhalis*; *Neisseria meningitidis*; *Proteus vulgaris*; *Staphylococcus aureus*; *Staphylococcus epidermidis*; *Clostridium perfringens*; penicillin-resistant *S. pneumoniae*; vancomycin-resistant *Enterococcus* (VRE) and additional clinical isolates (≈33 strains total)	Complete inhibition observed for MRSA, VRE, PRSP and *Candida albicans*; Growth inhibition ranging from 40 to 100% across tested strains; study design did not distinguish bacteriostatic from bactericidal effects and was limited to controlled in vitro challenge conditions.

## Data Availability

The original contributions presented in this study are included in the article. Further inquiries can be directed to the corresponding authors.
